# Physical Disability Affects Women’s but Not Men’s Perception of Opposite-Sex Attractiveness

**DOI:** 10.3389/fpsyg.2021.788287

**Published:** 2021-12-07

**Authors:** Farid Pazhoohi, Francesca Capozzi, Alan Kingstone

**Affiliations:** ^1^Department of Psychology, University of British Columbia, Vancouver, BC, Canada; ^2^Department of Psychology, McGill University, Montréal, QC, Canada

**Keywords:** attractiveness, personality, empathy, physical disability, mate preference

## Abstract

Physical appearance influences our perceptions, judgments, and decision making about others. While the current literature with regard to the perceptions and judgments of nondisabled people’s attractiveness is robust, the research investigating the perceived physical attractiveness and judgments of physically disabled individuals is scarce. Therefore, in the current study, we investigated whether people with physical disabilities are perceived by the opposite sex as more or less attractive relative to nondisabled individuals. Our results, based on over 675 participants, showed a positive effect for women’s attractiveness ratings of men with physical disabilities, but not men’s attractiveness ratings of physically disabled women. Moreover, social desirability bias was positively associated with attractiveness ratings of physically disabled individuals, meaning those with higher tendency to be viewed favorably by others rated physically disabled individuals more attractive. Finally, our results revealed that attractiveness ratings of individuals with physical disabilities are positively associated with extroversion and empathy in both men and women, and positively with agreeableness and negatively with neuroticism in women. In conclusion, our study showed women rate men with physical disabilities as higher on attractiveness than nondisabled men, which is also influenced by their social desirability bias.

## Introduction

Physical appearance can influence our perceptions, judgments, and decision making about others. Such perceptions and impressions regarding attractiveness and personality can be made both from faces (Zebrowitz and Montepare, 2008) and bodies (Carr and Friedman, 2005; Puhl and Heuer, 2009; Hu et al., 2018) and they can form very rapidly (Willis and Todorov, 2006; Todorov et al., 2009; Pazhoohi et al., 2020). The effect of physical attractiveness can also influence perceptions of social and intellectual competence (Eagly et al., 1991), intelligence (Zebrowitz et al., 2002), and even one’s first impression as to whether an individual is trustworthy or a possible romantic partner (Fletcher et al., 2014; South Palomares and Young, 2018). It has also been shown that individuals will make systematic personality inferences merely from the shape of a person’s body (Hu et al., 2018), or nuanced changes in body postures and movements (Isbister and Nass, 2000; Koppensteiner and Grammer, 2011; Fink et al., 2012). In light of this plethora of evidence, it is not surprising to discover that perceptions of a person’s facial and bodily attractiveness can influence explicit and implicit behaviors ranging from whether they are offered a job (Tews et al., 2009) to whether they are identified as a potential partner (Saxton et al., 2006).

Studies of physical attraction, and theories of its importance to human evolution, have its roots in evolutionary biology and psychology (Symons, 1979; Barber, 1995; Gangestad and Scheyd, 2005). For instance, research has shown that facial symmetry serves as an indicator of healthy development and genetic quality and is considered more attractive and preferred (Little et al., 2011; Sugiyama, 2015). Another instance is the secondary sexual dimorphic characteristics in faces (e.g., larger jawbones and prominent cheekbones in men) and bodies (height, muscularity and large upper bodies in men, and hourglass body shapes including large and firm breasts, and lower ratios of waist to hip in women) that are indicators of health and genetic quality, and thereby mate value (Puts, 2010; Little et al., 2011; Sugiyama, 2015; Pazhoohi et al., 2020). Altogether, physical characteristics that signal health and genetic quality are preferred and considered attractive by men and women in a potential partner as they eventually contribute to survival and reproductive success of their progenies (Symons, 1979; Barber, 1995; Gangestad and Scheyd, 2005).

From the above it would seem to follow that individuals with physical disabilities will be considered less attractive. Indeed, an evolutionary psychological perspective suggests that noncontagious physical disfiguration and behavioral disabling conditions can activate cognitive disease-avoidance processes, a “false positive” signal detection and response to a threatening disease induced situation (Park et al., 2003). Surprisingly, however, this question of physical disability and attractiveness has not been tested directly. The majority of previous research and theories on attractiveness concern perceptions and judgments of nondisabled individuals, and the handful of studies that have been conducted with disabled individuals has tended to focus on the risk that they face with regard to discrimination in the job market and hiring (e.g., Premeaux, 2001; Graffam et al., 2002; Gouvier et al., 2003; Ameri et al., 2018). Moreover, the few studies that have focused explicitly on attractiveness have been concerned primarily with self-perception of one’s attractiveness (e.g., McCabe and Taleporos, 2003; Lease et al., 2007). The rare studies that have examined how other people (i.e., those of the opposite sex) perceive people with physical disabilities in terms of attractiveness, did so somewhat indirectly or used a biographical vignette. For instance, Fichten et al. (1991) examined thoughts and feelings about a sighted person dating someone with a visual impairment and found that sighted individuals reported being less comfortable and less likely to date individuals with visual impairments, potentially indirectly suggesting an effect of disability on perceived desirability and/or attractiveness (Fichten et al., 1991). Additionally, research has found interesting connections between perceptions of attractiveness and judgments of behavior or personality in disabled individuals. For example, it is reported that nondisabled people are more likely to think that a physically attractive person who has been seriously injured will need less time to recover and is more likely to be responsible for the accident (Bordieri et al., 1983). Similarly, more physically attractive disabled individuals are rated as having less pain or disability, but the personality of more attractive disabled individuals is rated more favorably than less physically attractive disabled individuals (LaChapelle et al., 2014). Using biographical vignettes, previous research has asked college students about the perceived attractiveness of individuals with and without disabilities while simultaneously describing the protagonist with positive achievements in activities based on community, athletics, school, and work (Man et al., 2006; Rojahn et al., 2008). This research found no difference between attraction ratings in disabled and nondisabled scenarios and suggested that “future research examining nondisabled persons’ perceptions of people with disabilities using vignettes and photographs should investigate whether showing a full picture of someone with a disability has any effect on attraction ratings” (Man et al., 2006).

Accordingly, the aim of the present study is to take an initial step toward the investigation of the perception of physical attractiveness in individuals with physical disabilities, by asking participants to rate attractiveness in opposite-sex individuals. Furthermore, previous research has shown an important role of raters’ personality characteristics on their openness toward disability-related issues and disabled individuals (e.g., García et al., 2005). For example, empathy has been reported to associate with increased positive attitudes toward people with disabilities (e.g., Crystal et al., 1999; Levett-Jones et al., 2017). Thus, we also examined raters’ personality traits and empathy. Finally, as individuals with disabilities are a vulnerable group and social desirability bias (SDB; participants’ tendency to answer questions in a way to be viewed favorably by others) might affect attitudes and values toward them, we also asked a subsample of participants to complete a social desirability bias questionnaire (Stöber, 2001; Stöber et al., 2002).

While the nature of this study is somewhat exploratory, the role of personality traits would seem to complicate the prediction derived from evolutionary biology and psychology that people with disabilities will be rated as less attractive. A competing hypothesis is that individuals with higher empathy, more openness to experience, higher extraversion, and lower neuroticism will have more positive attitudes toward physical disabilities and rate such individuals as more attractive. Moreover, as women generally report higher levels of personality traits (i.e., neuroticism, extraversion, agreeableness, and conscientiousness) and empathy (Eisenberg and Lennon, 1983; Schmitt et al., 2008), we expect sex differences to emerge. Note that all questionnaires were presented before the stimuli to minimize the effect that observing physically disabled individuals might have on the self-report scores.

## Materials and Methods

### Participants

Participants were recruited from Amazon Mechanical Turk workers located in the United States. A total of 677 individuals (271 men and 406 women) participated in this study and completed an online survey with monetary compensation. Men were aged between 18 and 85 years (*M* = 38.96, *SD* = 14.83) and women were aged between 18 and 79 years (*M* = 41.68, *SD* = 15.74). A total of 330 participants (48.7%) reported being married, and 11.7% reported being not married but in a relationship. Additionally, 25.3% reported being single, and 14.4% were either widowed, divorced, or separated. As for their highest educational degree, 25.8% had high school diploma, 7.8% had a post-secondary diploma, 44.8% had undergraduate degree, 19.9% had master’s or equivalent graduate degree, 0.7% had elementary school, and 0.9% of the participants had a PhD degree. A G*Power analysis for a 2 × 2 mixed-effects design indicated that 148 participants would be sufficient to detect a small to moderate effect size (*f* = 0.15, *β* = 0.95).

### Stimuli

**Figure 1:**
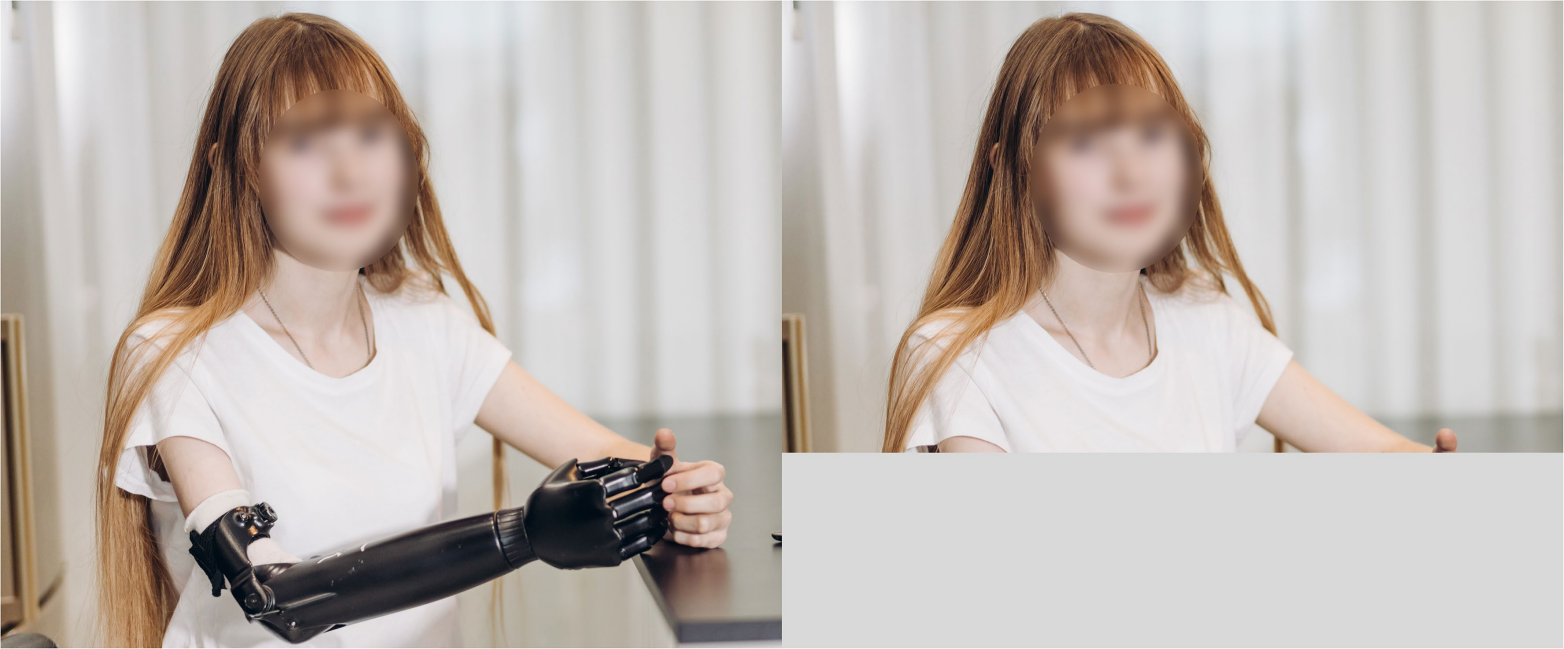
Example of stimuli with disability (left) and without disability (right).

Forty-four male and 44 female images with physical disabilities (e.g., amputated legs, amputated arms, either with prosthetics or without) were collected from the Internet. An extra version of the images was created by cropping the disability from the image, in order to create the visual impression that the individual does not have a physical disability (see Figure 1 as an example). For each sex, we created four sets of stimuli. Set A included 22 male images with physical disabilities, and set B consisted of the same 22 male images without a physical disability (i.e., the physical disability was cropped from the photo). Set C consisted of the other 22 male images with physical disabilities, and set D was composed of those images with the physical disability cropped from the images. Female participants were assigned to the group observing either sets A and C, or B and D. The same procedure was followed for creating the stimulus sets and conditions of the female images which were then displayed to the male participants. Note that in this way all the observers saw all the images of the opposite sex (22 with a physical disability and 22 without), but they never viewed an image of the same person more than once.

### Measures

#### Big Five Personality Traits

We used the Big Five Inventory-10 (BFI-10; Rammstedt and John, 2007) which is a 10-item short version of the Big Five model (Costa and McCrae, 1992). Participants were asked to rate their own personality on a 5-point Likert scale, with the results providing a measurement of the participants on five personality traits: agreeableness (sympathetic and warm vs. critical and quarrelsome), conscientiousness (dependable and self-disciplined vs. disorganized and careless), extraversion (extraverted and enthusiastic vs. reserved and quiet), neuroticism (anxious and easily upset vs. calm and emotionally stable), and openness (open to new experience and complex vs. conventional and uncreative).

#### Interpersonal Reactivity Index

The Interpersonal Reactivity Index (IRI) was used to measure participants’ empathy, defined as the “reactions of one individual to the observed experiences of another” (Davis, 1980). IRI is composed of four subscales, each made up of seven different items (a 28-item questionnaire). Participants are asked to rate on a 5-point Likert scale four separate aspects of empathy (Davis, 1980): Perspective Taking (the tendency to spontaneously adopt the psychological point of view of others), Fantasy (tendency to transpose themselves imaginatively into the feelings and actions of fictitious characters in books, movies, and plays), Empathic Concern (assessing feelings of sympathy and concern for unfortunate others), and Personal Distress (measuring feelings of personal anxiety and unease in tense interpersonal settings).

#### Social Desirability Scale

The Social Desirability Scale-17 (SDS-17; Stöber, 2001; Stöber et al., 2002) as a 16-items questionnaire was used (originally 17-item scale) and anchored on a 7-point Likert scale following recent recommendations (Larson, 2019). The scale provides a measurement of participant bias for answering questions in socially desirable ways (e.g., “I never hesitate to help someone in case of emergency” or “I always accept others’ opinions, even when they do not agree with my own”).

### Procedure

After consenting to participate in the study and answering our demographic questions (age, sex, sexual orientation, education, and marital status), participants completed the BFI-10 and IRI. In addition to the BFI-10 and IRI measures, a sample of participants (N = 392;131 men and 261 women) answered the SDS-17. The questionnaires including IRI and SDS were always presented before the stimuli to minimize the bias in empathy self-reports and avoid the effect of observing physically disabled individuals on the ratings.

After completing the questionnaires, male participants were then presented with two blocks of female stimuli, each block containing 22 images of females. One block contained images of 22 physically disabled women and the other block contained images of the 22 other women without their physical disabilities being shown. For female participants, the design was the same but the images were of males. The images within any given block were randomized, and the order of the blocks was randomized across participants. Participants were asked to rate each of the images on attractiveness (“How attractive do you find this person?”) on a 7-point Likert scale ranging from *not at all* (1) to *very* (7). Participants’ average of the ratings between the two groups of nondisabled stimuli was not significantly different [for female stimuli: *t*(133) = 0.55, *p* = 0.581; for male stimuli: *t*(201) = 0.78, *p* = 0.432], indicating that the nondisabled stimuli were well matched across the sexes.

### Statistical Analysis

A linear mixed model was conducted to investigate the effect of physical disability on the perceptions of attractiveness. Disability and Sex were added as fixed factors in the model, with Participants as a random factor. Analyses were conducted using RStudio version 1.4.1106, with lme4 version 1.1–21 and lmerTest version 3.1–0.

## Results

Results (*N* = 677) showed significant main effects of Disability and Sex, which were qualified with a significant Disability × Sex interaction (see Table 1 for details). *Post-hoc* analyses showed that women rated physically disabled men [*M* = 4.33, *SEM* = 0.07, 95% CI (4.19, 4.46)] more attractive than nondisabled men [*M* = 4.02, *SEM* = 0.07, 95% CI (3.89, 4.16), *p* < 0.001], while men did not rate physically disabled women [*M* = 4.97, *SEM* = 0.08, 95% CI (4.81, 5.14)] differently from nondisabled women on attractiveness [*M* = 4.96, *SEM* = 0.08, 95% CI (4.79, 5.12), *p* = 0.999].

**Table 1:** Effects for mixed model predicting attractiveness as a function of disability and sex.

			95% CI				
Effect	Estimate	*SE*	Lower	Upper	*df*	*t*	*p*
(Intercept)	4.57	0.05	4.47	4.67	675	87.3	<0.001
Disability (1-Yes, 2-No)	−0.16	0.03	−0.22	−0.10	675	−5.38	<0.001
Sex	0.79	0.10	0.59	1.00	675	7.57	<0.001
Disability * Sex	0.29	0.06	0.17	0.41	675	4.83	<0.001

### Social Desirability Bias

Another linear mixed model using the subsample of participants (*N* = 392) was conducted to investigate the effect of physical disability on the perceptions of attractiveness, including SDB as a covariate. Cronbach’s alpha for SDB was acceptable (*α* = 0.77). Disability and Sex were added as fixed factors in the model and SDB as a covariate, with Participants as a random factor. Results showed significant effect of SDB, as well as Disability and Sex, which similar to the previous analysis, were qualified by a significant Disability × Sex interaction (see Table 2 for details). The positive association of SDB indicates that both men and women were influenced by their social desirability bias when rating attractiveness. In other words, as participants’ tendency to be viewed more favorably by others increased, they also tended to rate the individuals as more attractive.

**Table 2:** Effects for mixed model predicting attractiveness as a function of disability and sex, with SDB as a covariate.

			95% CI				
Effect	Estimate	*SE*	Lower	Upper	*df*	*t*	*p*
(Intercept)	4.36	0.07	4.21	4.50	389	59.1	<0.001
Disability (1-Yes, 2-No)	−0.28	0.04	−0.36	−0.19	390	−6.46	<0.001
Sex	0.95	0.15	0.66	1.24	389	6.41	<0.001
SDB	0.54	0.10	0.35	0.72	389	5.55	<0.001
Disability * Sex	0.33	0.09	0.17	0.50	390	3.91	<0.001

*Post-hoc* analyses showed that women rated physically disabled men [*M* = 4.11, *SEM* = 0.09, 95% CI (3.93, 4.28)] more attractive than nondisabled men [*M* = 3.66, *SEM* = 0.09, 95% CI (3.49, 3.84), *p* < 0.001], while men did not rate physically disabled women [*M* = 4.88, *SEM* = 0.13, 95% CI (4.64, 5.13)] differently from nondisabled women on attractiveness [*M* = 4.78, *SEM* = 0.13, 95% CI (4.53, 5.02), *p* = 0.713].

An independent sample t-test was also conducted to compare SDB for men (*M* = 4.62, *SD* = 0.71) and women (*M* = 4.71, *SD* = 0.73), and no difference was found, *t*(390) = 1.06, *p* = 0.292.

### Correlation Analysis

Correlation analysis between ratings of the physically disabled stimuli only and personality traits as well as empathy was conducted using the full sample of participants (*N* = 677). Cronbach’s alpha for empathy aspects was acceptable (*α* > 0.72). Results for male participants showed that their attractiveness ratings of physically disabled women were positively associated with their extraversion personality, *r*(269) = 0.34, *p* < 0.001, 95% CI [0.23, 0.44]. For female participants, ratings of attractiveness of physically disabled men were positively associated with extraversion [*r*(404) = 0.23, *p* < 0.001, 95% CI (0.14, 0.32)] and agreeableness [*r*(404) = 0.15, *p* = 0.003, 95% CI (0.05, 0.24)], and negatively with neuroticism [*r*(404) = −0.12, *p* = 0.019, 95% CI (−0.21, −0.02)].

As for the empathy, male participants’ ratings of attractiveness of physically disabled stimuli only were positively associated with their self-report of empathic concern [*r*(269) = 0.29, *p* < 0.001, 95% CI (0.17, 0.39)], perspective taking [*r*(269) = 0.23, *p* < 0.001, 95% CI (0.11, 0.34)], and fantasy [*r*(269) = 0.31, *p* < 0.001, 95% CI (0.20, 0.41)]; while for female participants, attractiveness ratings of physically disabled men were associated with empathic concern [*r*(404) = 0.12, *p* = 0.018, 95% CI (0.02, 0.21)] and perspective taking [*r*(404) = 0.21, *p* < 0.001, 95% CI (0.12, 0.31)].

To explore whether SDB influences association between attractiveness ratings of physically disabled individuals and empathy, we applied a partial correlation analysis to the subsample of participants that answered the SDB survey (*N* = 392),^1^ thereby controlling for any SDB. The same results appeared between empathy and attractiveness for male participants empathic concern [*r*(129) = 0.18, *p* = 0.044], perspective taking [*r*(129) = 0.20, *p* = 0.026], and fantasy [*r*(129) = 0.21, *p* = 0.019], while only perspective taking remained significant for female participants [*r*(259) = 0.16, *p* = 0.010].

In sum, these results suggest that SDB partially explains the relationship between the empathy and attractiveness ratings for both men and women (as all the correlation coefficients are smaller when controlled for SDB). Moreover, the nonsignificant association between female participants’ empathic concern and their ratings when controlled for SDB indicates that their social desirability bias explains their empathy in relation to their positive attractiveness ratings of physically disabled men.

## Discussion

Previous research has considered physical attractiveness mainly from an evolutionary perspective using nondisabled individuals. However, for the first time, the current research investigated the perception of attractiveness in individuals of the opposite sex with physical disabilities. Personality traits, interpersonal empathic reactivity, and social desirability bias were also measured to test for the potential contribution of individual differences on perception. Our results revealed that women considered physically disabled men as more attractive than nondisabled men, while no difference was found for men’s attractiveness ratings of women as a function of physical disability.

Results of the analysis of a subsample that answered the social desirability scale showed a similar effect for men and women’s ratings, as well as a positive association with social desirability bias (SDB), indicating social desirability played a role in the ratings of attractiveness.

Collectively, the results show that women rate physically disabled men more attractive than nondisabled men and suggest that women’s tendency to inaccurately report on sensitive topics (e.g., judgment of physically disabled individuals), as was measured using SDB, was positively associated with their higher attractiveness ratings of men. This is in line with previous findings showing a positive attitude toward physical disabilities, as well as a sex difference in such an attitude, with women holding a more favorable attitude compared to men (Fonosch and Schwab, 1981; Olkin and Howson, 1994; Satchidanand et al., 2012). However, it should be noted that the previous studies mainly tested healthcare workers or school-age children’s attitudes toward physical disabilities (e.g., Ten Klooster et al., 2009; Satchidanand et al., 2012; Bustillos and Silván-Ferrero, 2013; Yorke et al., 2017), but not in an interpersonal attraction context. The finding that women rate disabled men more attractive is counterintuitive from an evolutionary perspective. From an adaptive perspective, the predication is that both men and women would consider nondisabled individuals more attractive compared to disabled individuals, as such characteristics might trigger disease-avoidance mechanism (Park et al., 2003). Our results suggest that individual differences in personality and empathy override the influence of such a mechanism in the perception of attractiveness.

Attractiveness ratings of physically disabled individuals were positively associated with extraversion personality in both men and women. The positive association of extraversion and attractiveness ratings is in line with previous research finding a positive relationship between extraversion personality and preference for attractive faces (Fink et al., 2005; Pound et al., 2007; Welling et al., 2009). However, our finding of a positive association between physical disability attractiveness ratings and extraversion suggests that individuals who happen to be more outgoing and energetic consider physically disabled individuals of the opposite sex as more attractive. For female participants, agreeableness (positively) and neuroticism (negatively) were associated with ratings of physically disabled male attractiveness. This suggests that more friendly and compassionate women consider physically disabled men more attractive, as do women who score lower on measures of neuroticism. To the best of our knowledge, this is the first investigation on the association between personality traits and attractiveness ratings of individuals with physical disabilities.

Similarly, no previous research has tested the relationship between personality traits and overall perception of individuals with physical disabilities. Clearly, more research is warranted to explore the relationship between personality traits of nondisabled individuals and their perception of people with physical disabilities.

As for measures of empathy, male and female participants’ ratings of attractiveness were positively associated with their empathic concern and perspective taking tendencies; though male’s attractiveness ratings were also associated with the fantasy aspect of empathy. In other words, in general, both male and female participants’ tendency to spontaneously adopt the psychological point of view of others and their ability in assess feelings of sympathy and concern for others was correlated with their attractiveness ratings of physically disabled individuals. While no previous study has tested the association of empathy and perception of attractiveness in physically disabled individuals, previous research has shown a similar positive relationship between empathy with regard to stigmatized groups (Batson et al., 1997, 2002). Moreover, a cross-cultural study provided evidence that individuals with a disability are often rated higher on perceived warmth, but not competence, than individuals without a disability (Cuddy et al., 2009). While we did not consider the characteristics of warmth and competence, our results show that women attributed more positive characteristics (i.e., attractiveness) to physically disabled individuals of the opposite sex.

## Future Remarks and Conclusion

In the current study, we were interested in investigating the effect of perception of attractiveness as a function of physical disability in an interpersonal attraction context, in which individuals consider and rate individuals of the opposite sex. However, future research can test for the potential interactions regarding the sex of the participants’ and the stimuli, as well as the effect that other variables, such as ethnicity and age, have on one’s attractiveness ratings. For example, it is an open question whether women view women with physical disabilities with the same positive bias as they view men, or participants view older individuals with physical disabilities similar as young people. Moreover, future research may assess the degree to which participants’ prior experiences in interacting with people with physical disabilities affect their ratings of attractiveness of physically disabled individuals. Previous research has shown that prior contact with individuals with disabilities can lead to more favorable and positive attitudes toward children, adolescents, and adults with disabilities (McDougall et al., 2004; Kalyva and Agaliotis, 2009; Seo and Chen, 2009; Perenc and Pęczkowski, 2018). Therefore, future research should consider such contact experiences on perception of attractiveness. Moreover, we used diverse categories of stimuli as the disability group (e.g., amputated legs, amputated arms, either with prosthetics or without), which might have influenced participants’ ratings. While our study lacks the statistical power to address this issue definitively, further studies could consider effect of each category separately on perception of attractiveness. Additionally, women’s attractiveness ratings of physically disabled men do not necessarily indicate they would actually want to date these men, a point that is reinforced by the social desirability data. Therefore, future research could investigate women’s level of interest in a short-term relationship as well as a long-term relationship with disabled men. It might be the case that women judge men with a disability hindered in their ability to secure and provide resources – a quality important in mate selection for women – thereby potentially reducing their interest in a long-term relationship with disabled men. Future research could also test the effect of attractiveness as a function of the cause of a disability. For example, pairing photos of a man’s disability with a story to explain the origin of the disability, either a story of an altruistic act (e.g., through military or police service) or a story about a reckless accident (e. g., car crash from driving drunk) might result in different attractiveness perception. For instance, in light of the fact that women find war heroes sexually attractive (Rusch et al., 2015), women may infer a disability resulting from an altruistic act as attractive. Finally, although we excluded the possibility of the visual images biasing the responses to the questionnaires by always presenting the questionnaires first, future research might choose to ensure that there was no effect of the questionnaires on the attractiveness ratings by inserting a distracting task between the two experimental procedures.

In summary, in the current study, we sought to examine if people with physical disabilities are perceived by the opposite sex as more or less attractive relative to nondisabled individuals. We also examined whether attractiveness ratings of physically disabled individuals are influenced by observers’ personality, empathy, and social desirability bias. Our results indicate that women rate men with physical disabilities as higher on attractiveness than nondisabled men. Such ratings were positively influenced by participants’ SDB, meaning those with higher tendency to be viewed favorably by others rated physically disabled individuals more attractive. Physical disability, however, does not appear to play a role in male perception of female attractiveness. Finally, our results reveal that ratings of individuals with physical disabilities are positively associated with extroversion and empathy in both men and women, and associated positively with agreeableness and negatively with neuroticism in women. These findings suggest that individual differences in personality and empathy can offset or override the influence of disease-avoidance mechanisms as predicted by an evolutionary perspective.

## Data Availability Statement

The raw data supporting the conclusions of this article will be made available by the authors, without undue reservation.

## Ethics Statement

The studies involving human participants were reviewed and approved by UBC’s Behavioral Research Ethics Board. The patients/participants provided their written informed consent to participate in this study.

## Author Contributions

FP: conceptualization, methodology, formal analysis, writing – original draft, and writing – review and editing. FC and AK: conceptualization, methodology, and writing – review and editing. All authors contributed to the article and approved the submitted version.

## Funding

This work was supported by a Killam Postdoctoral Research Fellowship awarded to FP, and grants to AK from the Natural Sciences and Engineering Research Council of Canada (2016-04319), and the Social Sciences and Humanities Research Council of Canada (435-2019-0749).

## Conflict of Interest

The authors declare that the research was conducted in the absence of any commercial or financial relationships that could be construed as a potential conflict of interest.

## Publisher’s Note

All claims expressed in this article are solely those of the authors and do not necessarily represent those of their affiliated organizations, or those of the publisher, the editors and the reviewers. Any product that may be evaluated in this article, or claim that may be made by its manufacturer, is not guaranteed or endorsed by the publisher.

## Footnotes

^1^All participants did not receive the SDB as its inclusion was suggested following a preliminary report of our study.
